# Theoretical Examination on the Chiral Separation Mechanism of Ibuprofen on Cellulose Tris(4-methylbenzoate)

**DOI:** 10.3390/molecules30173503

**Published:** 2025-08-26

**Authors:** Xiao Huang, Yuuichi Orimoto, Yuriko Aoki

**Affiliations:** Department of Material Sciences, Faculty of Engineering Sciences, Kyushu University, 6-1 Kasuga Park, Fukuoka 816-8580, Japan; huang.xiao.170@m.kyushu-u.ac.jp (X.H.); orimoto.yuuichi.888@m.kyushu-u.ac.jp (Y.O.)

**Keywords:** elongation method, molecular docking, cellulose tris(4-methylbenzoate), ibuprofen, complexation energy, interactions

## Abstract

The mechanism of separating the small chiral drug molecules on large soft polymers is essential in pharmaceutical science. As a case study, the differentiation mechanism of ibuprofen, (*R*,*S*)-2-(4-isobutylphenyl)propanoic acid, with cellulose tris(4-methylbenzoate) (CMB) as the chiral stationary phase (CSP) was investigated by combining the molecular docking simulation and multi-level layered terminal-to-center elongation (ML-T2C-ELG) method. Our results demonstrated that, based on the optimized geometry using the ML-T2C-ELG method, the complexation energy of *S*-ibuprofen with CMB obtained at B3LYP-D3(BJ)/6-311G(d) level is more negative than that of *R*-ibuprofen, which is caused by the greater hydrogen bonding and π-π stacking interactions between CMB and *S*-ibuprofen. The results are in line with the experimental observations of high-performance liquid chromatography (HPLC) that the retention time of *S*-ibuprofen on CMB is longer than that of *R*-ibuprofen. Moreover, the ML-T2C-ELG method was found to be valuable for optimizing the geometries of such flexible and large systems, which allows for a more accurate description of interactions between soft polymers and small molecules when coupled with the docking simulation. It is anticipated that this study can provide beneficial insights for future optical resolution mechanisms of other chiral drugs.

## 1. Introduction

Exploring the separation mechanism of chiral drugs is of considerable importance in the pharmaceutical field, owing to the commonly distinct biological behaviors of chiral drug enantiomers. Among various chiral drugs, ibuprofen is categorized as the 2-arylpropionic acid (2-APA) class with a chiral carbon center on its propionic side chain, resulting in two stereoisomers, namely *R*-ibuprofen and *S*-ibuprofen. It is a popular nonsteroidal anti-inflammatory drug to treat various diseases, owing to its good therapeutic performance in relieving pain, inflammation, and fever [1]. These symptoms are correlated with the generation of prostaglandins (PGs), in which the cyclooxygenase-1 (COX-1) and cyclooxygenase-2 (COX-2) enzymes play important roles [2]. Ibuprofen can reduce the production of PGs by suppressing the activities of these two enzymes, thereby alleviating the above-mentioned symptoms. Its commercial formulation is racemic, but its enantiomers differ in pharmacodynamics, pharmacokinetics, and toxicity [3,4]. The studies in experiment showed that *S*-ibuprofen exhibits much more pharmacological activity than *R*-ibuprofen, and it can reach a higher plasma concentration with the same clinical effectiveness and tolerance relative to racemic ibuprofen [5]. Significant improvements in its safety and efficacy could be expected if the product only includes the more active *S*-ibuprofen enantiomer [6]. Taking these into account, developing a single enantiomer drug should be carried out based on the chiral separation, which is considerably important for the pharmacokinetic and pharmacological analysis and quality control of enantiomers [7,8].

Regarding the chiral separation of enantiomers, high-performance liquid chromatography (HPLC) is a very effective and extensively used method, where the polysaccharide derivatives act as one of the most widely applied chiral stationary phases (CSPs) [9,10,11,12,13]. Among the polysaccharide-based CSPs, Okamoto and co-workers performed a systematic study to prepare and estimate a series of cellulose and amylose derivatives by modifying the substituents on the phenyl moieties to examine their potential as CSPs [14,15,16,17]. It was found that cellulose- and amylose-based derivatives are suitable to resolve various racemates, such as numerous aliphatic or aromatic compounds and chiral drugs, due to their remarkable chiral recognition, stability, and high sample loading capacity. The results indicated that the chiral recognition abilities of the above CSPs can be affected significantly when introducing different substituents on the different positions of the phenyl groups. For instance, in the cellulose benzoate derivatives, the introduction of a 4-substituted electron-donating group such as a methyl group can lead to a greater chiral recognition ability relative to that of a 2-substituted electron-withdrawing group such as a halogen [17]. This may be because the carbonyl groups of the cellulose benzoate derivatives exhibit a high electron charge density, which can be stabilized by the inductive effect of the methyl substituents [17]. Among various cellulose benzoate-based CSPs, cellulose tris(4-methylbenzoate) (CMB) bearing the methyl substituents on the phenyl groups presents an excellent enantioselectivity in the chiral separation of ibuprofen enantiomers [9]. In HPLC studies, chiral ibuprofen was effectively resolved on CMB, with *S*-ibuprofen having a longer retention time than *R*-ibuprofen [9].

Apart from the enantioselective recognition in experiments, molecular modeling, such as molecular dynamics (MD), molecular docking, and quantum chemical (QC) calculations, has been identified as a highly capable tool in elucidating the molecular-level chiral separation mechanism through the examination and analysis of interactions between CSPs and small chiral molecules. Given the large size and complexity of research systems in chiral separation, MD and molecular docking are widely applied to explore such chiral separation mechanisms [12,18,19,20]. QC calculations are generally unsuitable for large systems due to the huge computational cost, but they can be performed on relatively small systems to understand part of the mechanism [21]. For example, QC analyses of noncovalent O-H/Pt interactions indicated the remarkable energy differences between cisplatin and transplatin, offering great insights into their interactions with small- and large-scale biological molecules [22]. In view of these situations, it may be necessary to employ multiple methods together to achieve more comprehensive mechanistic insight, such as the combination of molecular docking simulation and QC calculations. The molecular docking simulation can be used to generate possible docking patterns. QC calculations can be employed to carry out geometry optimization, so that CSPs can interact with chiral molecules as much as possible. However, the large size and flexibility of CSP structures employed in chiral separations make it difficult to locate the global minimum geometries because of the presence of many local minima. Meanwhile, owing to the exponential computational complexity, massive resource requirements, and a lot of local minima, performing the full geometry optimization for such a large and complex system at a high level of theory is still challenging using the current QC calculations. Considering these circumstances, the elongation (ELG) method (see Section 3.1.1) constructs a computational framework to mimic the experimental polymer chain growth by adding the monomers one by one from one reaction terminal to the other one [23] at the ab initio level of theory, which can be used to study the molecular properties [24]. This method enables highly accurate calculations for the large one- and three-dimensional periodic and non-periodic systems with reasonable cost because it only calculates the active region, unlike the conventional QC method that concentrates on the whole system. A unique feature of this method lies in its stepwise progression, where each calculation step can use the results of the previous one, allowing flexible calculations to be repeated from any desired step using the stored intermediate data on disk. Based on the original ELG method, the developed ELG geometry optimization method has been proven to be valuable in geometry optimization of large-scale systems since it can achieve a good agreement with the results of conventional calculations [25]. The terminal-to-center (T2C) ELG method (see Section 3.1.2) was further developed [26] to initiate calculations from both reaction terminals toward the reaction center, supposing the active site is included in the center. Following the T2C-ELG method, the multi-level layered T2C-ELG (ML-T2C-ELG) method (see Section 3.1.3) was also established to enable various parts of the system to be handled at different computational levels. This ML-T2C-ELG method allows us to concentrate only on the essential central part of the system, which can be treated at a high computational level, like performing geometry optimization or using a large basis set. Therefore, the ML-T2C-ELG method could be applied to investigate the separation mechanism of chiral drugs on CSPs.

To isolate the pharmacologically active enantiomer of a chiral drug, it is essential to understand the theoretical mechanism of separating chiral drugs first. This work aims to elucidate the enantiomeric separation mechanism on the CSPs by analyzing the interactions between small chiral molecules (taking ibuprofen as an example) and large soft polymers (taking CMB as an example), based on the combination of molecular docking simulation and the ML-T2C-ELG method.

## 2. Results and Discussion

In the current work, there are three steps involved in exploring the separation mechanisms of the chiral molecule ibuprofen on CMB by examining and analyzing the interactions between CMB and ibuprofen. The first step is to predict the binding structures of ibuprofen with CMB using molecular docking simulations in preparation for subsequent QC calculations. The second step is to perform geometry optimization with the ML-T2C-ELG method on the essential central part, which includes the structures of ibuprofen and the CMB central unit (see Section 3). Here, the ML-T2C-ELG method is adopted because it can allow the high computational level to be applied only to the focused, crucial central part of the system rather than the entire system (see Section 3), thus ensuring that the relevant interactions between CMB and ibuprofen can be thoroughly reflected. The third step is to analyze the interactions between CMB and ibuprofen and evaluate the complexation energies based on the density functional theory (DFT) method (see Section 3.2). In this step, to properly assess the complexation energies of the studied systems, where the potential interactions such as hydrogen bonding and π-π stacking may exist and play a crucial role, employing DFT methods along with dispersion corrections is necessary.

**Figure 1:**
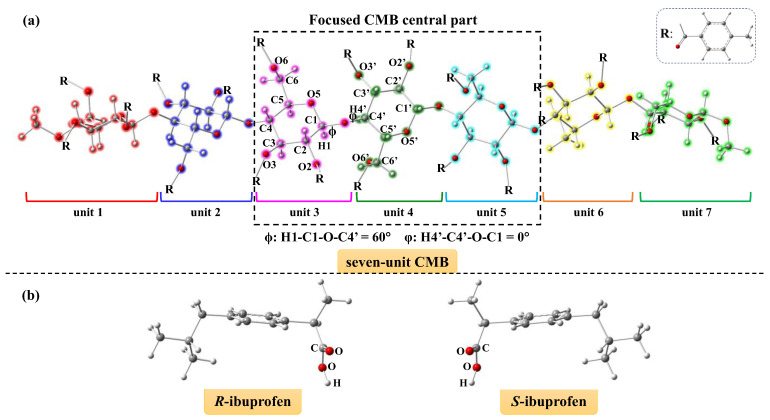
(**a**) Seven-unit CMB structure along with the key parameters, and (**b**) *R*-ibuprofen and *S*-ibuprofen structures optimized at B3LYP-D3(BJ)/6-31G(d) level for docking simulations.

In the first step of molecular docking simulation, the important parameters of CMB structure used in this work were derived from the study reported by Okamoto et al. [27]. Figure 1 displays the CMB structure with the atom notations and numberings (see Figure 1a), as well as the ibuprofen structures (see Figure 1b) that are optimized for docking simulations. The CMB structure with seven units marked in different colors, as shown in Figure 1a, was adopted in the docking simulations, allowing us to concentrate on the CMB central three units that could interact with the ibuprofen. Each unit of the CMB structure consists of a glucose unit from cellulose and three 4-methylbenzoate groups attached to it, as shown in Figure 1a.

### 2.1. Binding Structures of Ibuprofen with CMB Based on Docking Simulation

**Figure 2:**
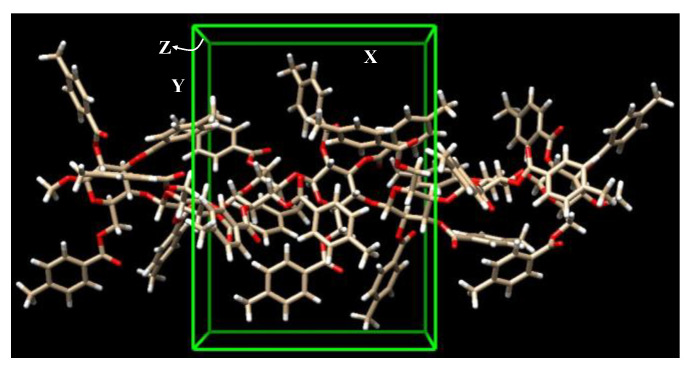
CMB structure with seven units used in the docking simulations. The ibuprofen scan range around the central three units of CMB is marked with a green-colored box.

Figure 2 displays the seven-unit CMB structure used in the molecular docking simulations, and the associated scanning regions of ibuprofen around CMB, where the central three units of the CMB structure are included along the *x*-axis range. For a clear comparison, *R*-ibuprofen and *S*-ibuprofen should be situated in the same space of CMB. To ensure this point, the ibuprofen scanning range is constrained to the CMB central three units, as presented in the green-colored box of Figure 2.

**Figure 3:**
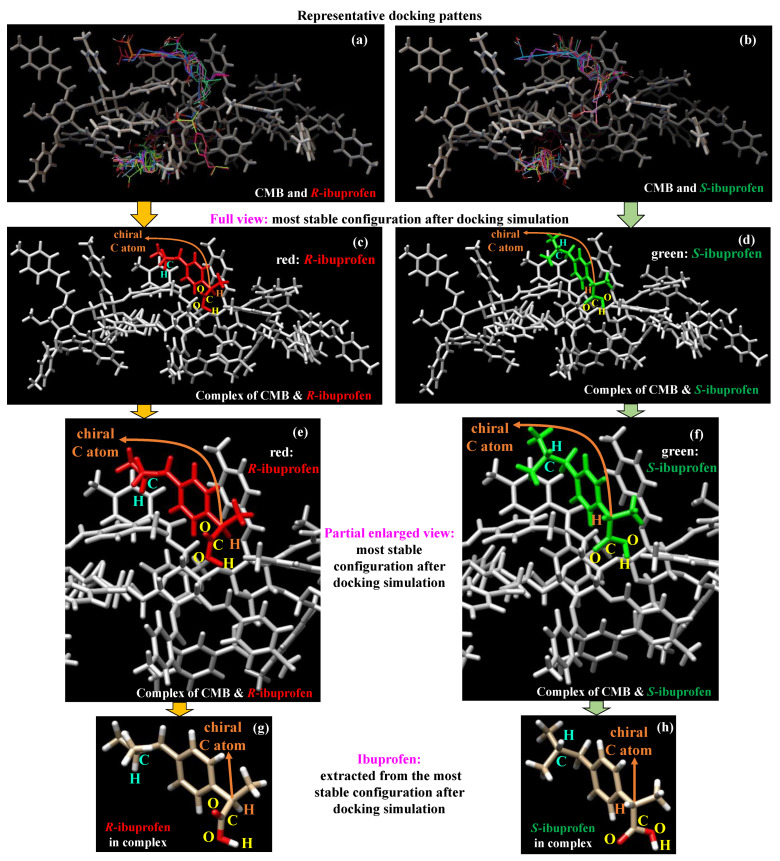
Representative docking pattens for CMB (in gray) with (**a**) *R*-ibuprofen and (**b**) *S*-ibuprofen (in color) with each showing fifty configurations of ibuprofen around the CMB structure; the (**c**,**d**) full and (**e**,**f**) partial enlarged views of the most stable binding structure between CMB (in gray) and *R*-ibuprofen (in red) or *S*-ibuprofen (in green) after docking simulation; and the (**g**) *R*-ibuprofen and (**h**) *S*-ibuprofen extracted from the most stable binding structure.

To present the binding structures after docking simulation distinctly, Figure 3 displays the typical configurations of *R*-ibuprofen (see Figure 3a) and *S*-ibuprofen (see Figure 3b) around the CMB structure, the most stable binding structure between CMB and *R*-ibuprofen (see Figure 3c,e) or *S*-ibuprofen (see Figure 3d,f) in full and partial enlarged views, respectively, and the corresponding ibuprofen (see Figure 3g,h) extracted from the most stable configuration. To clarify, only the top 50 configurations presenting the lowest interaction energies are illustrated in an overlap manner, as shown in Figure 3a,b. Upon analysis, it is found that these configurations for *R*-ibuprofen and *S*-ibuprofen are predominantly located at three different positions around the CMB structure, indicating the existence of interaction preferences between them. Due to the three-fold helical nature of the CMB structure [28], these three binding positions are regarded as equivalent to each other. From these configurations, the one exhibiting the lowest interaction energy was selected for each ibuprofen enantiomer, as displayed in Figure 3c,d. The selected configurations demonstrate that *R*-ibuprofen (see Figure 3c) and *S*-ibuprofen (see Figure 3d) are placed in the same space of CMB, thus facilitating a meaningful comparison of their binding behaviors. However, only the configuration with the lowest energy as a representative structure for subsequent analysis has certain limitations, such as neglecting conformational diversity, potentially overlooking alternative binding modes, and so on. Considering these situations, a systematic conformational search or clustering analysis could be performed in the following work.

As shown in Figure 3c–f, apart from the visually similar π-π stacking interactions, there are three major different places in the selected binding structures, presenting the geometric differences between *R*-ibuprofen (see Figure 3c,e) and *S*-ibuprofen (see Figure 3d,f) after docking simulation. (1) For *R*-ibuprofen, the H atom bound to -CH- within the -CH_2_CH(CH_3_)_2_ group is oriented toward the inner side of the CMB pocket, while for *S*-ibuprofen, it faces the outer side. (2) The H atom attached to -CH- in the -CHCH_3_(COOH) group points toward the internal orientation of the pocket for *R*-ibuprofen. Conversely, it points to the external orientation for *S*-ibuprofen. (3) For *R*-ibuprofen, the O atom of C=O in the -COOH group faces the exterior and the H atom of -OH points to the interior of the binding pocket, whereas for *S*-ibuprofen, both are positioned toward the interior. The opposite orientations in (1) and (2) may result in similar interactions for *R*-ibuprofen or *S*-ibuprofen with CMB. However, two similar orientations toward the binding pocket for *S*-ibuprofen in (3) may facilitate stronger hydrogen bonding interactions for *S*-ibuprofen with CMB. These results indicate that relative to *R*-ibuprofen, *S*-ibuprofen exhibits a more favorable conformation after docking, which may facilitate a better fit within the binding pocket.

However, due to the rotational flexibility of ibuprofen and the rigid treatment of the large soft CMB structure in the docking simulation, some key interactions may not be properly represented, thus leading to considerable limitations of the above docking-generated results. In order to capture the comprehensive interactions between CMB and ibuprofen as much as possible, the selected configuration mentioned above was subsequently taken as the starting structure for the next step of geometry optimization with the ML-T2C-ELG method.

### 2.2. Comparison of Geometries Before and After Geometry Optimization

**Figure 4:**
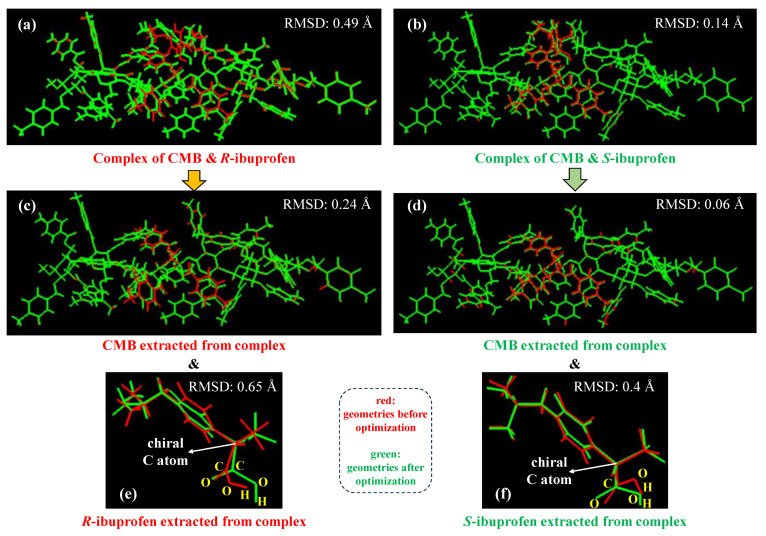
Comparison of geometries before (in red) and after (in green) geometry optimizations with the ML-T2C-ELG method by examining the RMSD values for the (**a**,**b**) whole system, (**c**,**d**) CMB, and (**e**,**f**) ibuprofen isolated from the complex.

Based on the selected binding structures from docking simulations as mentioned above, geometry optimizations using the ML-T2C-ELG method are performed to explore the detailed interactions between CMB and ibuprofen. The calculations of the ML-T2C-ELG method start from both terminals to the center simultaneously. Each terminal contains a 2-mer oligomer, which is called the starting cluster. In the final step, the interactive region includes the CMB central three units (units 3–5, see Figure 1) and ibuprofen, where the unit 4 of CMB along with ibuprofen are optimized while the adjacent unit 3 and unit 5 of CMB are fixed, aiming to minimize the boundary effects between the frozen and active regions [25] (see Section 3.2). The corresponding computational details are described in the following Section 3.2.2. To evaluate the structural changes introduced by geometry optimization with the ML-T2C-ELG method, Figure 4 displays the comparison of geometries before and after geometry optimizations. The geometric differences are assessed by examining the values of root mean square deviation (RMSD). Figure 4 is produced using VMD 1.9.3 [29] software, which is also utilized to obtain the RMSD values.

As presented in Figure 4, the geometric variations before and after geometry optimizations are compared for three parts: (1) the whole complex including CMB and ibuprofen (see Figure 4a,b), (2) the CMB extracted from the complex (see Figure 4c,d), and (3) the ibuprofen isolated from the complex (see Figure 4e,f). Here, the different reference frames in RMSD analysis were adopted for CMB and ibuprofen because the focus is on the internal conformational changes individually rather than the comparison of the relative motion of ibuprofen with CMB. The advantage of such a treatment is that the impact of the overall drift of the complex can be eliminated, enabling a greater focus on local flexibility like the movement of ibuprofen itself.

At first, the RMSD values for the whole complex, including CMB and ibuprofen, are calculated to present the overall geometric changes. As for the whole complex, the RMSD values are 0.49 Å and 0.14 Å for the complexes of CMB and *R*-ibuprofen and CMB and *S*-ibuprofen, respectively, as illustrated in Figure 4a,b. These values suggest that the geometries exhibited some changes after geometry optimization using the ML-T2C-ELG method relative to those before geometry optimization. In other words, the geometric alterations via the examination of these RMSD values indicate that the ELG method could be a useful tool to perform geometry optimization on such large and soft research systems possessing weak interactions, which was also confirmed in the earlier study on the analysis of interactions between DNA bulge and different ligand molecules [26]. In addition, the RMSD value of 0.49 Å for the CMB and *R*-ibuprofen complex is larger than that for the CMB and *S*-ibuprofen complex. It demonstrates that, compared to the geometry before geometry optimization, it undergoes greater geometric alterations upon binding for the complex of CMB and *R*-ibuprofen than CMB and *S*-ibuprofen. The larger RMSD values for the CMB and *R*-ibuprofen complex may be because *R*-ibuprofen has an improper spatial conformation, less structural match with the binding pocket, or potential steric hindrance caused by the orientations of functional groups. It means that *R*-ibuprofen may present lower structural stability and binding adaptability, which can cause a larger geometric deviation from the initial geometry after optimization.

Subsequently, to identify which part mainly leads to the geometric changes in the entire complex, the corresponding individual structures of CMB (see Figure 4c,d) and ibuprofen (see Figure 4e,f) are isolated from the whole complex, and their respective RMSD values are examined. As depicted in Figure 4c–f, the RMSD values for CMB and *R*-ibuprofen in the CMB and *R*-ibuprofen complex are 0.24 Å and 0.65 Å, respectively, and those for CMB and *S*-ibuprofen in the CMB and *S*-ibuprofen complex are 0.06 Å and 0.4 Å, respectively. The larger RMSD values for ibuprofen in complex indicate that the geometry of ibuprofen in complex is larger than that of CMB in complex when performing geometry optimization with the ML-T2C-ELG method. It may be because, during the geometry optimization process, the smaller ibuprofen molecule is more likely to experience conformational changes because of its relative flexibility caused by non-covalent interactions with CMB, whereas the larger CMB molecule remains relatively rigid due to the geometric restrictions at both terminals. These results indicate that the overall geometric alterations are mainly attributed to the geometric changes in ibuprofen.

### 2.3. Analysis of Interactions Between CMB and Ibuprofen

Following the geometries after geometry optimizations using the ML-T2C-ELG method, the interactions of CMB with ibuprofen are then systematically investigated. First, the complexation energies between CMB and ibuprofen are examined based on the geometries after geometry optimization with the ML-T2C-ELG method. They are obtained by calculating the single-point energies and basis set superposition error (BSSE) with the DFT method using the Gaussian 16 [30] program package. The details about computations are shown in Section 3.2.3. Second, to better explore the interactions, the atoms contributing to the interactions of ibuprofen with CMB along with the corresponding distances are analyzed to illustrate the interaction patterns between them.

To understand the interactions between CMB and ibuprofen, Table 1 displays the complexation energies between CMB and *R*-ibuprofen and *S*-ibuprofen, respectively. Both the negative complexation energies demonstrate that the attachment of ibuprofen to CMB can lead to the stabilization of the entire system. As shown in Table 1, the complexation energy for the CMB and *S*-ibuprofen complex is −33.0 kcal/mol, which is lower by 2.8 kcal/mol than that (−30.2 kcal/mol) of the CMB and *R*-ibuprofen complex. The lower complexation energy indicates that *S*-ibuprofen can interact with CMB more strongly than *R*-ibuprofen. The results are consistent with the experimental observations, where the retention time of *S*-ibuprofen on CMB is longer than that of *R*-ibuprofen [9]. Although the retention order matching alone cannot completely capture quantitative agreement, this study concentrates on the qualitative consistency by verifying the correct retention order as an initial step, given that comparable experimental separation factors α and retention time differences were unavailable.

To evaluate the relevance of the 2.8 kcal/mol energy difference on chiral separation between *R*-ibuprofen and *S*-ibuprofen, the computational uncertainties, particularly the BSSE, should be considered. As displayed in Table 1, the BSSE of the CMB and *R*-ibuprofen and CMB and *S*-ibuprofen complexes are about 7.9 and 8.5 kcal/mol, respectively. The difference in BSSE correction is about 0.6 kcal/mol, which accounts for about 21% of the total 2.8 kcal/mol energy difference, indicating that the BSSE is not negligible. The qualitative trend of this energy difference is expected to maintain significance when more rigorous BSSE corrections or the larger basis sets are applied because the same computational level is adopted for both *R*-ibuprofen and *S*-ibuprofen systems. Moreover, this energy difference obtained based on the single-point calculations of the optimized geometries is a deterministic value within the chosen theoretical level and basis set, and thus no statistical uncertainty from replicates is available. In addition, the previous molecular docking studies on chiral separation [20,31] indicated that the greater energy difference between enantiomers can lead to higher enantioselectivity in chromatographic experiments. According to the estimated energy differences in the studies of the chiral separation mechanism using the docking simulation [20,31], the energy difference of 2.8 kcal/mol calculated in the current study could be sufficient to result in the chiral separation observed in the experiment.

To clarify the connection between the calculated energy difference and experimental results, the free energy difference between enantiomers can serve as a bridge, which can be evaluated via the relation of Δ*G* = RT lnα [19,32]. Δ*G* is the free energy difference, and α is the separation factor that can be obtained from the chromatographic experiment. As reported in the study of chromatographic experiments for the separation of ibuprofen enantiomers on CMB, the separation factor α is evaluated to be 1.18 using mice plasma samples [9]. Based on Δ*G* = RT lnα and the separation factor α = 1.18 [9], Δ*G* is estimated to be about 0.1 kcal/mol. However, it is inappropriate to compare this experimental value with our calculational result, because the former corresponds to the free energy difference with temperature effect while the latter is the complexation energy difference without temperature effect. Although the former is smaller than the latter, they exhibit consistency with each other qualitatively. Here, the comparison between them was intended to highlight the qualitative preference for one complexation mode over another, rather than to reproduce the experimental free energies quantitatively. Furthermore, various experimental factors such as the solvation will be considered using the classical MD simulations in our future work to estimate the corresponding free energy difference, expecting to establish the relationships between the calculational and experimental results.

**Figure 5:**
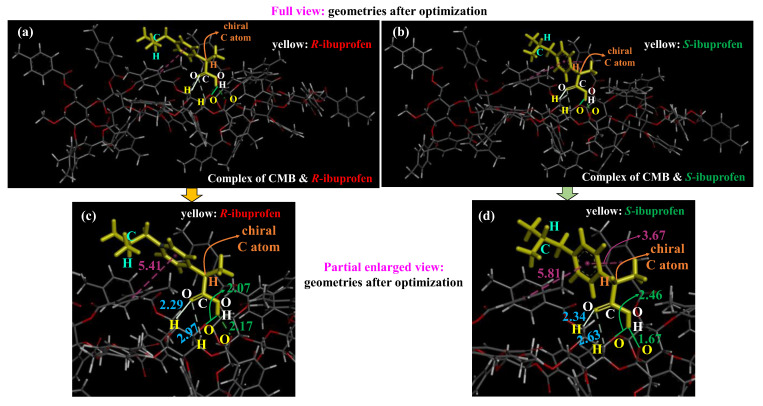
Geometries after optimizations using the ML-T2C-ELG method for the complexes of CMB and *R*-ibuprofen and CMB and *S*-ibuprofen, shown in (**a**,**b**) full and (**c**,**d**) partial enlarged views. The distances (Å) of atom pairs between CMB and ibuprofen are marked in partial enlarged view.

To further explore the detailed interactions between CMB and ibuprofen, the geometries of the CMB and ibuprofen complex after geometry optimization with the ML-T2C-ELG method, presented in both full and partial enlarged views, are shown in Figure 5. The interaction patterns, the corresponding distances and angles between two phenyl rings within the atom pairs between CMB and ibuprofen are displayed in Table 2.

As shown in Figure 5 and Table 2, there are two types of interactions between CMB and ibuprofen. One is the hydrogen bonding interaction, and the other is the π-π stacking interaction. For the hydrogen bonding interaction, the corresponding distances of atom pairs in the CMB and *R*-ibuprofen complex are 2.29 Å, 2.97 Å, 2.07 Å, and 2.17 Å, while those in the CMB and *S*-ibuprofen complex are 2.34 Å, 2.63 Å, 2.46 Å, and 1.67 Å, respectively. Compared to those in the CMB and *R*-ibuprofen complex, the former three hydrogen bonding interactions are almost equivalent to the interactions of CMB with *S*-ibuprofen, where the largest difference in the corresponding distance is 0.39 Å (2.46 Å − 2.07 Å), as displayed in Table 2. Notably, for the interactions of CMB with ibuprofen, the last hydrogen bonding distance is 1.67 Å between CMB and *S*-ibuprofen, while it is longer at 2.17 Å between CMB and *R*-ibuprofen. It indicates that this hydrogen bonding between CMB and *S*-ibuprofen is much stronger than that between CMB and *R*-ibuprofen. It may be explained by the presence or absence of steric hindrance caused by the orientation of the -CH- hydrogen atom within the -CHCH_3_(COOH) group pointing into the binding pocket. In the CMB and *R*-ibuprofen complex (see Figure 5a,c), the inside orientation of the -CH- hydrogen atom toward the binding pocket leads to steric hindrance that prevents the -COOH group from approaching the binding pocket tightly via hydrogen bonding. In contrast, in the CMB and *S*-ibuprofen complex (see Figure 5b,d), the outside orientation of the -CH- hydrogen atom toward the binding pocket results in the absence of steric hindrance that enables the -COOH group to bind more closely to the binding pocket via hydrogen bonding. However, hydrogen bonding strength here was evaluated only based on the distances within atom pairs, which may impose limitations. To resolve this, charge and population analyses will be performed in the following studies. As for the π-π stacking interaction, T-shaped π-π stacking interaction is included in both the CMB and *R*-ibuprofen complex and CMB and *S*-ibuprofen complex, with the distances of 5.41 Å and 5.81 Å, respectively, as shown in Figure 5 and Table 2. To thoroughly clarify the strength of the π-π interaction, the angle between the two phenyl ring planes is displayed in Table 2. An angle between the two phenyl planes of 90° in the CMB and *S*-ibuprofen complex, compared to 122.2° in the CMB and *R*-ibuprofen complex, indicates a stronger T-shaped π-π interaction in the former. However, a parallel π-π stacking interaction with a distance of 3.67 Å was observed in the CMB and *S*-ibuprofen complex, whereas it was absent in the CMB and *R*-ibuprofen complex. In addition, the angle between two phenyl rings of 165.3° in the CMB and *S*-ibuprofen complex indicates a great parallel π–π interaction. These results suggest that the π-π stacking interactions between CMB and *S*-ibuprofen exhibit a greater strength than between CMB and *R*-ibuprofen. In the CMB and *R*-ibuprofen complex, the parallel π–π stacking is absent because *R*-ibuprofen adopts an unsuitable spatial conformation and has a poor structural fit with the binding pocket. However, in the CMB and *S*-ibuprofen complex, *S*-ibuprofen conformation may be well aligned with the binding pocket, supporting a parallel π–π stacking interaction. Therefore, according to the analysis of hydrogen bonding and π–π stacking interactions, the CMB and *S*-ibuprofen complex exhibits larger interactions than the CMB and *R*-ibuprofen complex. Relative to the results of the CMB and *R*-ibuprofen complex, the more negative complexation energy for the CMB and *S*-ibuprofen complex, as discussed above, can be explained in terms of the stronger interactions between CMB and *S*-ibuprofen.

Overall, compared to the results for *R*-ibuprofen, the stronger interactions of *S*-ibuprofen with CMB lead to a greater negative complexation energy for *S*-ibuprofen. In addition, the ML-T2C-ELG method can effectively carry out geometry optimization on such a large and flexible research system. Combining the molecular docking simulations with the ML-T2C-ELG method can investigate the detailed interactions between CMB and small molecules.

## 3. Method and Computational Details

### 3.1. Method

#### 3.1.1. ELG Method

**Figure 6:**
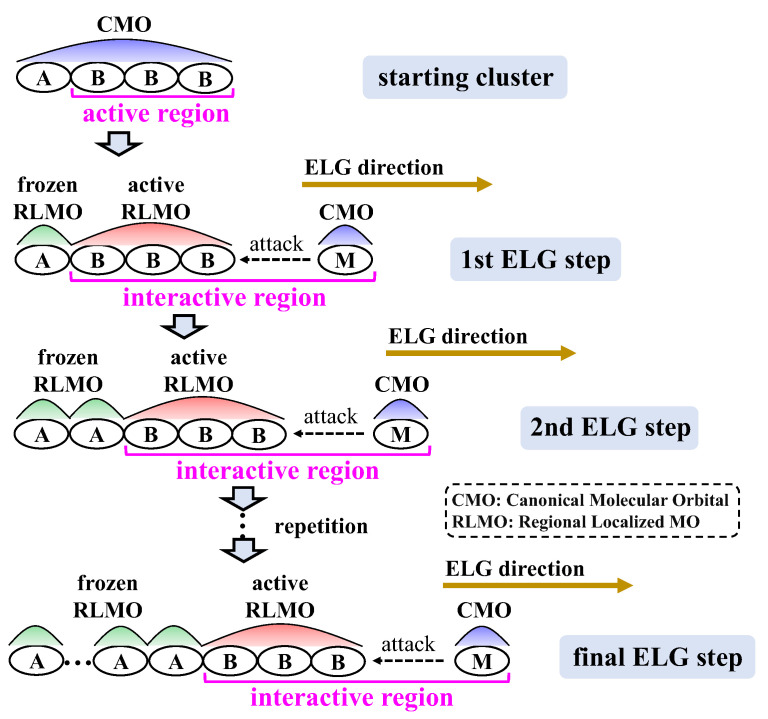
Procedure of the ELG method.

The ELG method built a computational framework to simulate the experimental process of polymer chain growth in the ab initio framework, which is systematically described in our earlier studies [23,24], and its procedure is shown in Figure 6. First, the canonical molecular orbitals (CMOs) that are delocalized across the entire cluster are achieved through the calculation of the starting cluster. Next, the CMOs are converted into regional localized MOs (RLMOs) distributed across the frozen A and active B regions, enabling the progression of calculations. B region corresponds to the interactive side of the molecular chain, whereas A region is situated at the non-reactive side far away from the interactive side. Afterwards, the attacking monomer (M) engages with the propagating terminal of the active B region in the starting cluster. The CMOs obtained in the current step are further localized to generate the updated frozen and active RLMOs for the subsequent ELG step. The above-outlined localization and elongation steps are alternately executed in a stepwise manner, extending the polymer chain to the desired length.

#### 3.1.2. T2C-ELG Method

**Figure 7:**
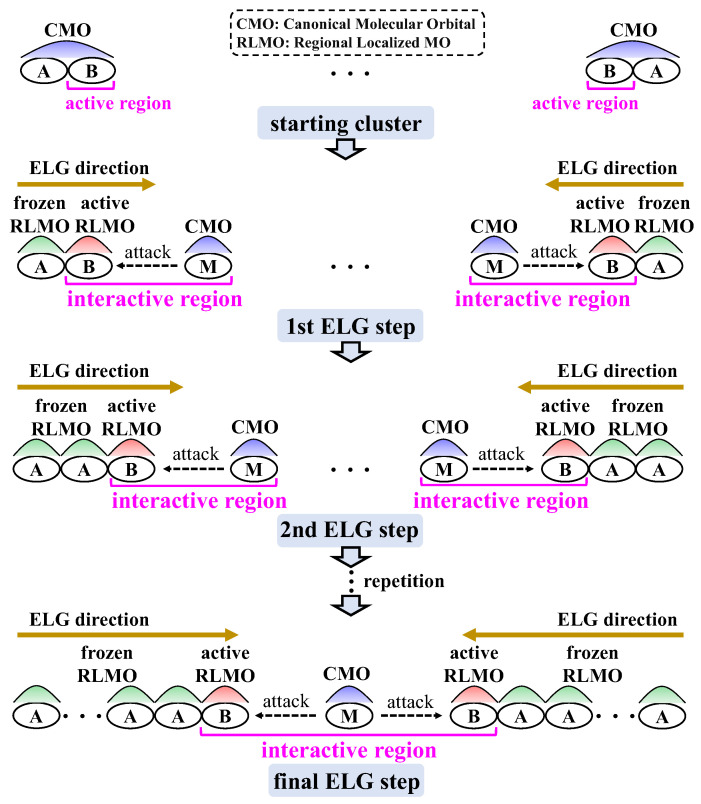
Procedure of the T2C-ELG method.

Based on the ELG method as mentioned above, the T2C-ELG [26] method was also developed within the ab initio theoretical framework, and the corresponding procedure is shown in Figure 7. With respect to the T2C-ELG method, the corresponding calculations of the starting cluster initiate at both terminals at the same time. For each terminal, the CMOs are localized in frozen or active RLMOs, which are situated in frozen A or active B region, respectively. Both active B regions interact with two monomers (M) inside simultaneously, followed by the repeated localization and elongation steps until the systems are linked to the final system.

#### 3.1.3. ML-T2C-ELG Method

**Figure 8:**
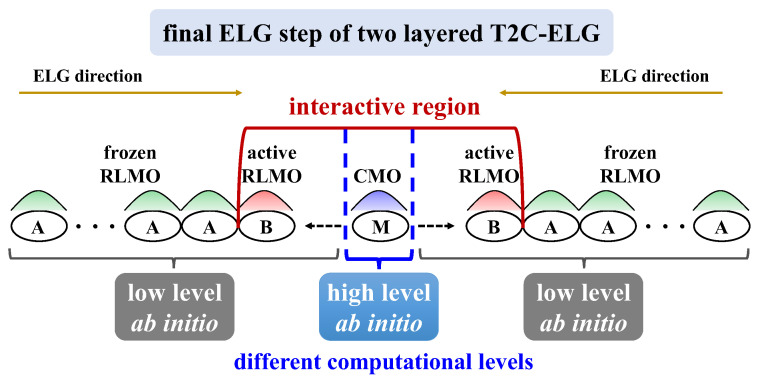
Computational levels applied to the final ELG step of the two layered T2C-ELG procedure.

According to the above T2C-ELG method, the ML-T2C-ELG [26] method was further developed under the ab initio level of theory, in which the multi-computational levels can be applied to the different parts of the research systems. This method ensures that the higher computational level can be used only in the essential part, while the lower computational level is used in the remaining parts. The higher or lower computational level mentioned here corresponds to a larger or smaller basis set, the inclusion or exclusion of electron-correlation effects, the implementation or omission of geometry optimization, respectively, and so on. To illustrate these situations, Figure 8 displays the different computational levels applied in different parts, taking the final ELG step of the two-layered T2C-ELG procedure as an example. As depicted in Figure 8, in the final ELG step, the central M part included in the interactive BM region adopts a higher computational level, whereas the B parts next to the M part use the lower computational levels. Such a treatment makes it possible to target the key reaction part of large research systems with high accuracy and reasonable cost, especially for the systems composed of large flexible polymer and small ligand molecules held together by non-covalent interactions. The main benefit of this treatment is that the electronic structures obtained from low-level computations can be stored and reused, enabling the calculations to be repeated only for high-level computations when introducing a new ligand.

### 3.2. Computational Details

#### 3.2.1. Docking Simulation

The molecular docking between CMB and ibuprofen was conducted using Autodock Vina [33]. The initial structures of ibuprofen were obtained by pre-optimization with a molecular mechanics (MM) force field employing the GaussView 6.1.1 program [34]. The structures of *R*-ibuprofen and *S*-ibuprofen optimized at B3LYP [35,36,37]-D3(BJ)/6-31G(d) level by the Gaussian 16 program package were used as the starting geometries for the docking simulations. Herein, B3LYP-D3(BJ) was used to optimize the ibuprofen structures because it could provide reliable results for this kind of organic molecule [38,39].

The GaussView 6.1.1 program was used to illustrate the CMB and ibuprofen structures (see Figure 1) before the docking simulations. As for the results of the docking simulations, UCSF Chimera 1.16 [40] and AutoDockTools-1.5.7 [41] were applied to show the binding structures (see Figure 3). The Biovia Discovery Studio Client 2018 was adopted to visualize the interactions between CMB and ibuprofen (see Figure 5).

#### 3.2.2. Geometry Optimization

**Figure 9:**
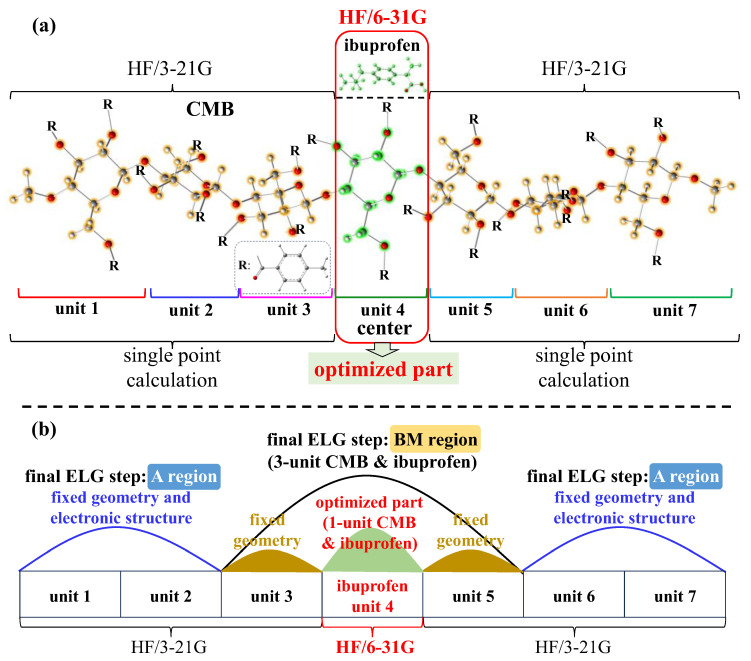
(**a**) The used basis sets and (**b**) situations of the final ELG step during geometry optimization of the CMB and ibuprofen complex using the ML-T2C-ELG method.

Derived from the selected docking-generated structures, the geometry optimizations were conducted employing the ML-T2C-ELG method incorporated in the GAMESS program package [42]. Figure 9 displays the detailed computational conditions for geometry optimization with the ML-T2C-ELG method. During the geometry optimizations, HF/6-31G was applied to the optimized part, which includes the CMB central unit (unit 4) and entire ibuprofen structure (in red-colored box), whereas HF/3-21G was used for the remaining parts to perform the single point calculations, as presented in Figure 9a. Here, these two basis sets were used during the calculations, since this study is at a preliminary stage and the main purpose is to confirm that such a system, including large polymers and small ligands, can be handled with different basis sets assigned to the significant central part and the remaining parts, respectively. During the geometry optimization, the optimization and SCF convergence criteria were set to 1.0 × 10^−3^ Hartree/Bohr and 1.0 × 10^−5^ Hartree, respectively. In this work, the starting cluster included two units (unit 1 and unit 7; see Figure 9) in the frozen A region and another two units (unit 2 and unit 6; see Figure 9) in the active B region with the ML-T2C-ELG method. As shown in Figure 9b, in the final step, the geometry optimization was performed on the CMB central unit (unit 4) and ibuprofen, with the adjacent unit 3 and unit 5 fixed, to reduce the coupling between the frozen A and active BM regions [25]. However, such treatment could restrict the relaxation of the surrounding units, which may lead to local distortions in the truncated region. Additionally, the constrained geometry may prevent the accommodation of ibuprofen, introducing minor artificial strain and slight deviations in the computed relative energies. Based on the previous work [26], this truncation could yield the reliable relative energies for such host–guest complexes.

#### 3.2.3. Complexation Energy Calculation

Based on the geometries after geometry optimization with the ML-T2C-ELG method, the single-point calculations and BSSE correction were further carried out in the gas phase at B3LYP-D3(BJ)/6-311G(d) level using the Gaussian 16 program package to obtain the complexation energy between CMB and ibuprofen. An ultrafine integration grid and the default SCF convergence criterion were employed. The counterpoise method [43] was adopted for BSSE correction, in which the orbitals and DFT grid points are placed on the ghost atoms.

A two-step calculation is included to obtain the complexation energy. First, geometry optimizations were performed at a low computational level to reduce computational cost, which is a common practice for large systems. Subsequently, single-point energy calculations were carried out at a higher computational level to improve the accuracy of electronic energies. Combining these two steps is widely adopted in computational studies to balance efficiency and accuracy. For instance, the smaller and larger basis sets were applied to conduct geometry optimization and to evaluate the energy, respectively [44,45]. However, due to a great improvement in the computational level (such as DFT functional and basis set) used in single point calculations relative to that in geometry optimization, it may affect the relative energies and other aspects. To minimize such effects, in the following work, the higher computational level will be employed for geometry optimization, and the effects on relative energies and others will be addressed.

## 4. Conclusions

Molecular docking simulation and the ML-T2C-ELG method were combined to investigate the separation mechanism of *R*-ibuprofen and *S*-ibuprofen on CMB. The results indicate that the complexation energy of the CMB and *S*-ibuprofen complex is lower by 2.8 kcal/mol than that of the CMB and *R*-ibuprofen complex, owing to the stronger interactions of *S*-ibuprofen with CMB, aligning well with the observation of the chromatographic experiment. There are two types of interactions between the atom pairs of CMB and ibuprofen: hydrogen bonding and π-π stacking interactions. Relative to the results of the CMB and *R*-ibuprofen complex, stronger hydrogen bonding and parallel π-π stacking interactions exist in the CMB and *S*-ibuprofen complex. It means that the complex of CMB and *S*-ibuprofen can be more stabilized by the above two interactions compared to the CMB and *R*-ibuprofen complex. In addition, the combination of molecular docking simulation and the ML-T2C-ELG method provides a valuable way to explore such separation mechanisms of chiral molecules on large polymer systems. The findings of this work are expected to offer critical mechanistic guidance to the separation of other small chiral drugs in the future and to be one reason that *S*-ibuprofen exhibits much more pharmacologically active behavior than *R*-ibuprofen.

## Figures and Tables

**Table 1:** Complexation energies for CMB with *R*-ibuprofen and *S*-ibuprofen based on the single point calculation and BSSE with the DFT method employing the Gaussian 16 program, respectively.

Ligand	*E_Total* (Complex) *^a^*	*E_Total* (CMB) *^b^*	*E_Total* (Ligand) *^c^*	*E* (BSSE)	Complexation Energy *^d^*
	(a.u.)	(a.u.)	(a.u.)	(kcal/mol)	(kcal/mol)
*R*-ibuprofen	−13,149.2976297	−12,492.3431564	−656.8938190	7.9	−30.2
*S*-ibuprofen	−13,149.2949832	−12,492.3403913	−656.8883585	8.5	−33.0

*^a^* Single point energy for the whole complex of CMB and ligand. *^b^* Single point energy for the CMB structure extracted from the complex. *^c^* Single point energy for the ligand structure extracted from the complex. *^d^* Complexation energy = *E_total* (complex) − *E_total* (CMB) − *E_total* (ligand) + *E* (BSSE).

**Table 2:** Interaction types and the distances (Å) of atom pairs between CMB and ibuprofen based on the geometry after optimization with the ML-T2C-ELG method.

Atom Pairs		Interaction Type	Distance (Å)		Angle (°) Between Two Phenyl Rings	
Ibuprofen	CMB		*R*-Ibuprofen	*S*-Ibuprofen	*R*-Ibuprofen	*S*-Ibuprofen
(C=O) O	H-C	O···H-C	2.29	2.34		
(C=O) O	H-C	O···H-C	2.97	2.63		
O-H	O (pyranose)	O-H···O	2.07	2.46		
O-H	O (O=C)	O-H···O	2.17	1.67		
phenyl	phenyl	T-shaped π-π	5.41	5.81	122.2	90
phenyl	phenyl	parallel π-π	— *^a^*	3.67	— *^b^*	165.3

*^a^* The distance is not detected in the software of Biovia Discovery Studio Client 2018. *^b^* The angle is not detected in the software of Biovia Discovery Studio Client 2018.

## Data Availability

The data presented in this work are available in the article.

## Abbreviations

The following abbreviations are used in this manuscript:

CMB	cellulose tris(4-methylbenzoate)
ELG	elongation
T2C-ELG	terminal-to-center elongation
ML-T2C-ELG	multi-level layered terminal-to-center elongation
HPLC	high-performance liquid chromatography
2-APA	2-arylpropionic acid
PGs	prostaglandins
COX-1	cyclooxygenase-1
COX-2	cyclooxygenase-2
CSPs	chiral stationary phases
MD	molecular dynamics
QC	quantum chemical
DFT	density functional theory
RMSD	root mean square deviation
BSSE	basis set superposition error
CMOs	canonical molecular orbitals
RLMOs	regional localized molecular orbitals
M	monomer
MM	molecular mechanics
